# Two-stage laparoscopic resection of colon cancer and metastatic liver tumour

**DOI:** 10.4103/0972-9941.15245

**Published:** 2005-03

**Authors:** Yukio Iwashita, Atsushi Sasaki, Toshifumi Matsumoto, Kohei Shibata, Masafumi Inomata, Masayuki Ohta, Seigo Kitano

**Affiliations:** Department of Surgery I, Oita University Faculty of Medicine, Oita 879-5593, Japan

**Keywords:** Colorectal cancer, metastatic liver tumour, laparoscopic hepatectomy, laparoscopy-assisted colectomy

## Abstract

We report herein the case of 70-year-old woman in whom colon cancer and a synchronous metastatic liver tumour were successfully resected laparoscopically. The tumours were treated in two stages. Both postoperative courses were uneventful, and there has been no recurrence during the 8 months since the second procedure.

## INTRODUCTION

Laparoscopy is used as minimal access surgery for colorectal cancer and liver tumours.[1] Herein, we describe a case of colon cancer and synchronous liver metastasis in which both tumours were treated laparoscopically.

## CASE REPORT

**Figure 1a:**
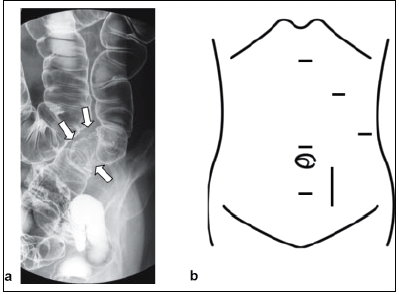
Barium enema shows an encircling tumour in the sigmoid colon (arrows), 1b: Port sites for laparoscopy assisted sigmoid colectomy

A 70-year-old woman was admitted to our hospital with a diagnosis of cancer of the sigmoid colon. Barium enema showed an encircling mass in the sigmoid colon (Figure 1). Computed tomography (CT) revealed a low-density area in the left lateral segment of the liver (Figure 2). Liver function was normal, and the preoperative carcinoembryonic antigen level was 6.2 ng/ml (normal range, < 5 ng/ml). The patient had no history of abdominal surgery. We planned a two-stage procedure for the patient because it was considered to be a highly invasive treatment for this elder patient when both sigmoid colectomy and hepatectomy were performed simultaneously. In addition, interval hepatic resection for synchronous metastases of colorectal cancer, with a routine waiting period of 4-6 months, was recommended to improve the patient selection.[2] Therefore, laparoscopic sigmoid colectomy was performed first. With the patient in the supine position, pneumoperitoneum of 8 mmHg was established, and trocars were placed in the right upper and lower abdomen (Figure 1). After the sigmoid colon was mobilized from the surrounding tissues, a skin incision (6 cm) was made in the left lower abdomen. The sigmoid colon was exteriorized and resected through the skin incision after appropriate barrier protection of the wound edges was ensured. Operation time was 372 min, and estimated blood loss was 70 ml. The postoperative course was uneventful, first flatus was recognized on day 2, solid diet was started on day 3, the patient was discharged and directly went home on postoperative day 11.

**Figure 2a:**
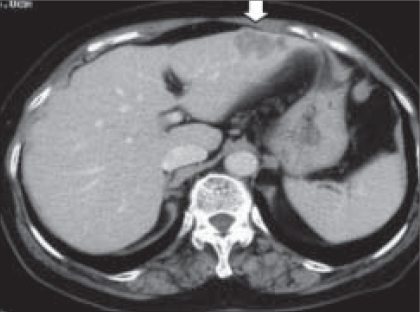
Computed tomographic scan shows a metastatic liver tumour (arrow)

**Figure 2b:**
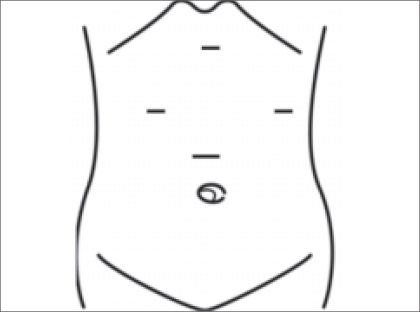
Port sites for laparoscopic partial hepatectomy

5 months after the first operation, CT scan revealed that the slight enlargement of the liver tumour (25 mm to 37 mm in diameter), but new lesions were not observed (Figure 2). We performed laparoscopic partial S2/3 hepatectomy. After CO_2_ insufflation with a pressure of 8 mmHg, inspection of the peritoneal cavity revealed no remarkable adhesion. Four trocars were placed in the upper abdomen, as shown in Figure 2. The falciform, left triangular, and coronary ligaments were dissected, and the left hepatic lobe was mobilized. Hepatic resection was performed with an endoscopic autosuture stapler (EndoGIAII, US Surgical, Norwalk, CT, USA) under lower pneumoperitoneum pressure to prevent gas embolism. The resected specimen was removed from the port site, which was enlarged to 3 cm in the supraumbilical area with an EndoCatchII device (US Surgical). Operation time was 167 min, and blood loss was 20 ml. The postoperative course was uneventful and the patient was discharged and directly went home on day 14. She is currently doing well, with no evidence of disease recurrence during the 8 months since the procedure.

## DISCUSSION

Laparoscopic surgery has become a standard technique for the treatment of benign diseases. Further development of instruments and techniques has made it possible to apply laparoscopic surgery to malignant diseases.[3] To our knowledge, this is the first report of laparoscopic resection of both primary tumour and metastatic liver tumour. In comparison to conventional surgery, laparoscopic surgery is beneficial with respect to short-term outcome, including earlier recovery and less pain. Our research in a murine model has shown that laparoscopic surgery is advantageous for gastrointestinal malignancies due to reduced impairment of systemic and intraperitoneal cell-mediated immune responses.[4] Although a comparison of long-term outcomes between laparoscopic and conventional surgeries for advanced colon cancer has been recently published,[5] there have been no reports of randomized trials of laparoscopic hepatectomy in patients with metastatic liver tumours. Future study is needed to further evaluate the usefulness of laparoscopic hepatectomy.
